# Unveiling the molecular mechanism of self-healing in a telechelic, supramolecular polymer network

**DOI:** 10.1038/srep32356

**Published:** 2016-09-01

**Authors:** Tingzi Yan, Klaus Schröter, Florian Herbst, Wolfgang H. Binder, Thomas Thurn-Albrecht

**Affiliations:** 1 Experimental Polymer Physics, Institute of Physics, Martin Luther University Halle-Wittenberg, Halle 06120, Germany; 2 Chair of Macromolecular Chemistry, Institute of Chemistry, Martin Luther University Halle-Wittenberg, Halle 06120, Germany

## Abstract

Reversible polymeric networks can show self-healing properties due to their ability to reassemble after application of stress and fracture, but typically the relation between equilibrium molecular dynamics and self-healing kinetics has been difficult to disentangle. Here we present a well-characterized, self-assembled bulk network based on supramolecular assemblies, that allows a clear distinction between chain dynamics and network relaxation. Small angle x-ray scattering and rheological measurements provide evidence for a structurally well-defined, dense network of interconnected aggregates giving mechanical strength to the material. Different from a covalent network, the dynamic character of the supramolecular bonds enables macroscopic flow on a longer time scale and the establishment of an equilibrium structure. A combination of linear and nonlinear rheological measurements clearly identifies the terminal relaxation process as being responsible for the process of self-healing.

The recovery of material properties after application of stress and rupture - often called ‘healing’ - is an essential property of living systems. The transfer of this ability to synthetic materials has become a major research topic in materials science in recent years^1^,^2^,^3^. Especially appealing are concepts where self-healing is an intrinsic property of the material. This can be accomplished by introducing dynamic bonds into the load carrying molecular structure of the material. In case of damage these dynamic bonds will break and subsequently reestablish, thereby restoring the mechanical strength of the material. For this process a high molecular mobility is needed, which makes soft matter systems especially suited for the design of self-healing materials^4^,^5^,^6^,^7^,^8^. Besides weak covalent bonds (such as eg. nitroxide/carbon bonds^9^, or disulfide-bonds^10^), noncovalent reversible bonding systems such as hydrogen-bonds in rubbery materials^5^,^11^, metal/ligand complexes in biomimetic systems^12^,^13^,^14^), pi/pi-stacking^15^ or electrostatic interactions in ionomers^16^ have been exploited to generate materials with self-healing properties. The load carrying structure itself typically consists of a scaffold or network surrounded by a mobile matrix allowing for exchange and repair of the constituents. For intrinsic self-healing at room temperature without external stimulus bonds with a highly dynamic character are required. The dynamic character of the bonds is normally linked to small association constants, leading to low mechanical strength and stiffness^17^. A prominent example are solvent containing gels like hydrogels. They often display a very good ability to heal fracture but they are typically too soft and weak for application as structural parts. But combined with phase segregation and high network densities in bulk materials, the mechanical properties can be strongly improved also for very weak hydrogen-bonding interactions^18^,^19^,^20^.

Although the the relation between molecular relaxation processes and self-healing was analyzed in theoretical studies^8^, attempts to experimentally elucidate the molecular processes responsible for self-healing in such networks have been less successful. As a starting point well controlled and well characterized network structures are desirable^21^. This is often difficult to achieve for established self-healing systems. E. g. in the classic example of supramolecular rubbery networks from small molecules^5^, the crosslink density is hard to control and the network structure is not in equilibrium^22^. In case of ionomers, the polar, ionic groups along the main chain aggregate and build up clusters acting as dynamic reversible crosslinks. But the phase separated structure of these systems is usually not well known and difficult to characterize^16^, as there is a hierarchy of aggregates from ion pairs to multiplets to ionic clusters. In ref. 23 indirect evidence was obtained that the time scale of self-healing is related to the time scale of molecular relaxation processes, but for the investigated systems an unequivocal assignment of broadly distributed, macroscopic relaxation processes to their molecular origin was difficult and the direct comparison to the self-healing process was hampered by the fact that self-healing required an intermediate high-temperature step leading to an additional thermally induced dissolution of the aggregates constituting the network.

We here employ a self-assembled reversible bulk network formed by aggregation and hydrogen-bonding of telechelic polymers which avoids these difficulties. Small angle X-ray scattering and rheological measurements show that a well-defined equilibrium network forms, made up from supramolecular assemblies. Due to the chemical design the different relaxation processes in the material are well separated and exhibit different activation energies which makes it possible to explore the relation between equilibrium dynamics and the kinetics of modulus recovery after non-linear deformation and yielding. The results clearly show that healing is connected to the network relaxation.

## Results

**Figure 1:**
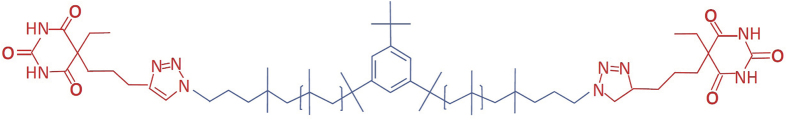
Chemical structure of telechelic Polyisobutylene endfunctionalized with barbituric acid.

**Figure 2:**
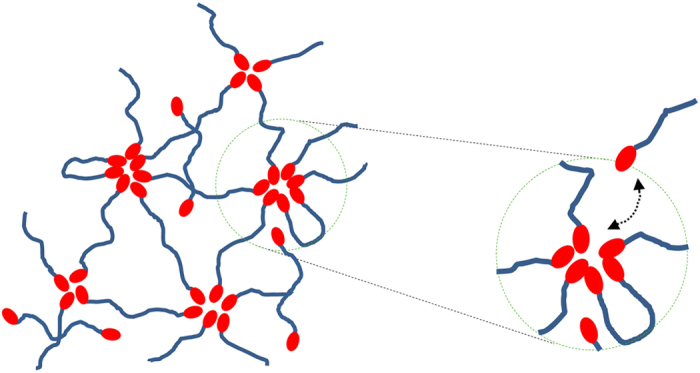
Schematic representation of the dynamic network formed by the telechelic PIB. The crosslinks are aggregates formed by the barbituric acid carrying end groups (red), which dynamically exchange between bound and unbound states. A certain fraction of free chains coexists with the aggregates.

Fig. 1 shows the model sample used for this study, telechelic polyisobutylene (PIB) chain with barbituric acid groups at both chain ends. The molecular weight (14 kg/mol) is comparable to the critical molecular weight of PIB, being on the one hand small enough to avoid strong effects by entanglements, on the other hand large compared to the molecular weight of the barbituric acid groups. As we will show below the functionalized chain ends assemble in well-defined aggregates forming a dynamic supramolecular polymer network as defined in ref. 24. This network structure and the exchange dynamics of a chain end is schematically depicted in Fig. 2.

### Structure and dynamics of reversible network in equilibrium

**Figure 3:**
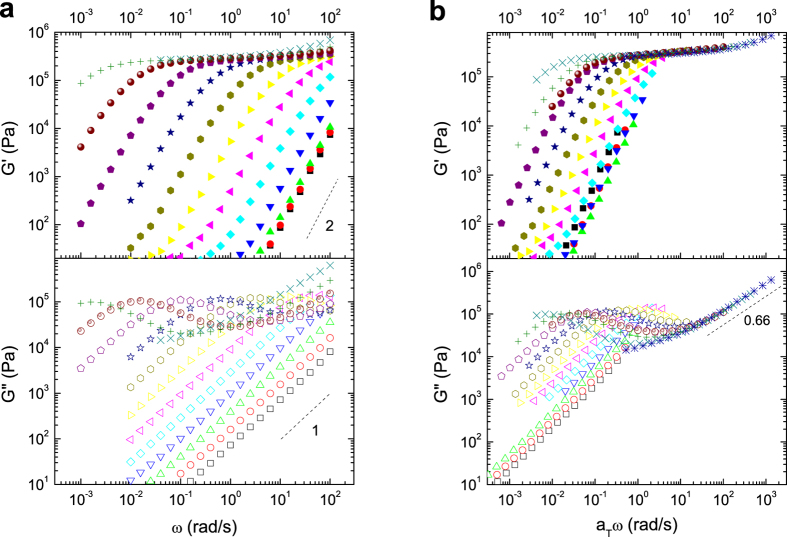
Linear rheology. (**a**) Real and imaginary part of the complex shear modulus v.s. angular frequency at different temperatures (from 10 to 120 °C from left to the right in 10 K steps) and (**b**) master curve for the real and imaginary part of the complex shear modulus v.s. scaled angular frequency at a reference temperature of 25 °C.

The formation of a reversible network structure can be proven by linear rheology. Fig. 3(a) shows the real (*G*′) and imaginary (*G*″) part of the dynamic shear modulus in dependence on frequency, measured for several temperatures. One notices a broad elastic plateau in the real part of the modulus. Due to the rather low molecular weight of the polymer, entanglements as an explanation for the plateau can be excluded. Also direct elastic interactions between aggregates can be excluded because of the low volume fraction of the aggregates (see scattering results below). Only an elastic network of bridging chains remains as an explanation. Left of the plateau at low frequencies typical terminal flow behavior shows up at all temperatures. The real and imaginary part of the modulus follow the typical slope of two, respectively one on a double-logarithmic scale. The onset of terminal flow depends on temperature and shifts towards higher frequencies with increasing temperature. Terminal flow proofs the dynamic nature of the bonds within the network, contrary to a covalent network which would prevent the sample from flowing. Finally, at high frequencies and best visible for the low temperature data the modulus further increases with a slope around one half. This is the typical signature for internal chain dynamics as described by the Rouse model^25^. To separate the temperature dependence of chain dynamics and the terminal relaxation we constructed a masterplot as it is usually done for samples following time-temperature-superposition. Contrary to the usual approach in literature, we did not try to overlap the data for the telechelic polymer directly and discuss the shift factors afterwards^26^, but rather followed a procedure suggested in ref. 27 and constructed a masterplot of the complex shear modulus based on the shift factors of PIB homopolymer representing the temperature dependence of the chain modes. These shift factors were determined by independent measurements of a PIB homopolymer without endfunctionalization.

Fig. 3(b) shows the resulting master curve. There is a perfect overlap of the chain modes at high frequencies. As expected, these relaxation processes and their temperature dependence are not much affected by the end groups. On the contrary, the terminal flow region does not overlap at all. This relaxation process shows a totally different temperature dependence, i.e. it is not controlled by relaxation processes of the PIB chain but by the separate dynamics of the network based on end group exchange. Hence in general, both processes can be controlled independently by the choice of the polymer backbone and the functional groups. The choice of PIB with its known small shift factor turns out to be very advantageous here as it enables a clear distinction between the temperature dependence of chain modes and the terminal relaxation. Other examples from literature with for instance poly(ethyl acrylate) as backbone lead to rather similar shift factors for both contributions, resulting in seemingly good overlap in the master curve over the whole range of temperatures^28^ which makes a clear discrimination of both contributions difficult.

**Figure 4:**
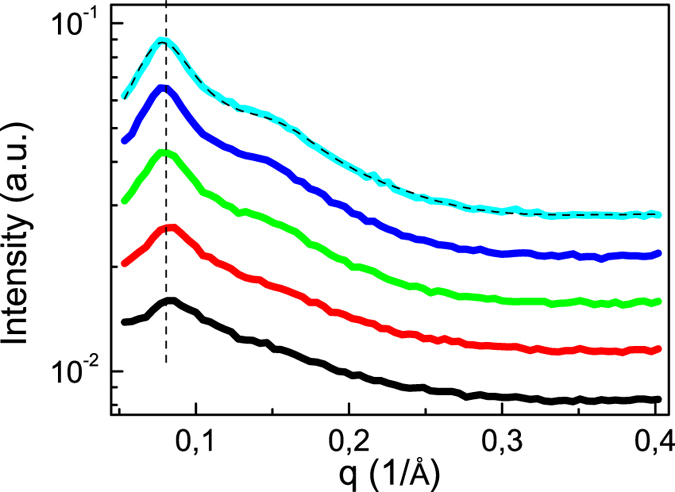
Small angle scattering results on sample PIB14K-BA2. Scattering intensity at a series of temperatures (120 °C; 80 °C; 40 °C; 0 °C; −40 °C; from bottom to top). The data are shifted vertically. A model curve based on a Percus-Yevick structure factor is shown for the data measured at the lowest temperature.

It remains to be shown that the crosslinking points of the networks are aggregates of chain ends. This fact follows from the results of small angle x-ray scattering (SAXS). Fig. 4 shows the corresponding scattering intensity vs. scattering vector *q* over a broad temperature range (−40 to 120 °C). The telechelic polymers can be considered as strongly asymmetric triblock copolymers. As it was shown before for monofunctional PIB chains with hydrogen-bonding endgroups, the scattering signal and the underlying structure can be understood in terms of the well-investigated structure formation in block copolymers^27^. Such systems form disordered spherelike micelles over an extended temperature range with an equilibrium of free chains and aggregates formed by the chain ends (depicted in red in Fig. 2). The large scattering signal in the range of small q is caused by these aggregates. The peak position corresponds to the average distance *d* between the aggregates, *d* = 2*π*/*q*_*peak*_, and the shoulder can be attributed to the form factor of the aggregates. With increasing temperature the fraction of free chains increases, leading to a loss of correlation between the aggregates and a weaker and broader peak in the scattering intensity. A truly disordered melt of individual chains with functional groups would only cause a correlation hole effect with a much weaker peak. This result excludes the prevalence of long, linear chain assemblies^29^, which otherwise could also lead to transient entanglements and a corresponding elastic plateau in the shear modulus. The fact that the peak position is nearly temperature independent indicates, that the mean distance between the aggregates or their number density stays the same over a broad range of temperatures. The actual value of *d* (8.1 nm) is a bit smaller than the calculated end-to-end distance of a Gaussian chain (9.7 nm) for PIB of that molecular weight^30^, both ends of a single chain will therefore with a high probability be contained in two separate aggregates. This result supports the suggested structure of a highly connected supramolecular polymer network as schematically depicted before in Fig. 2.

A quantitative analysis of the scattering data has been undertaken using the Perkus-Yevick model to calculate the structure factor^27^. The corresponding hard-sphere interaction is of course a strong simplification for the real interaction potential which should contain a long-range, attractive contribution due to bridging chains and a repulsive interaction, preventing interpenetration of neighboring, brush-like coronas of the aggregates. In fact, the simple, effective hard sphere potential is able to reproduce the most important features of the scattering data as found before for a similar system^27^. A model fit assuming spherical aggregates and a Percus-Yevick structure factor to the scattering data (shown for the lowest temperature) gives a nearly temperature independent aggregate size of about 1,3 nm, corresponding to about 20 chain end groups per aggregate. Approximate information about the relation between free and aggregated chains can be obtained from a comparison of the thus calculated aggregation number to the number of chains ends available in the volume per aggregate. The latter value can be estimated to be around 60, assuming a similar packing density of the micelles as in a bcc-structure^27^; i.e. at room temperature about one third of the chain ends are part of an aggregate, the others belong to dangling ends or free chains.

### Nonlinear rheological properties and self-healing

**Figure 5:**
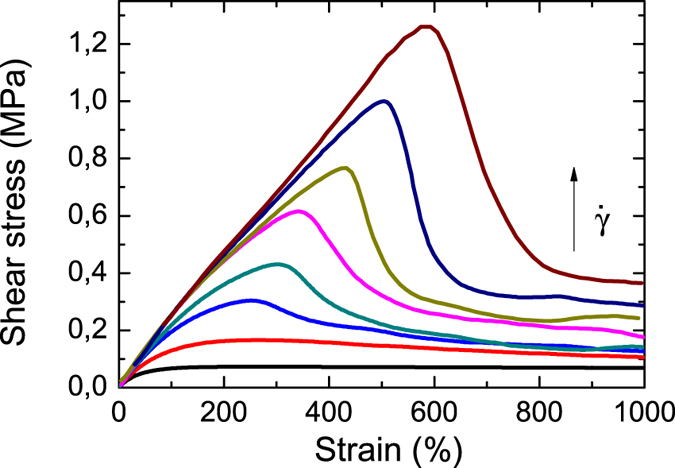
Startup shear measurements for PIB14K-BA2. Stress strain curves at 25 °C for different shear rates (*Wi* = 0.33 to 1000).

**Figure 6:**
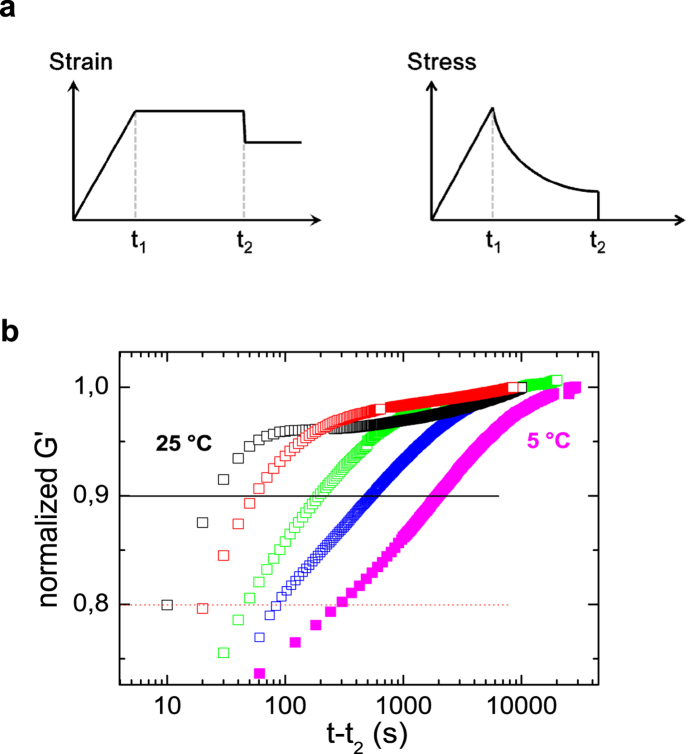
Self-healing experiment. (**a**) Schematic representation of the time dependence of strain and stress during the self-healing experiment. (**b**) Resulting normalized storage modulus during recovery for sample PIB14K-BA2 at different temperatures (25 °C, 20 °C, 15 °C, 10 °C, 5 °C) as a function of time *t* − *t*_2_.

**Figure 7:**
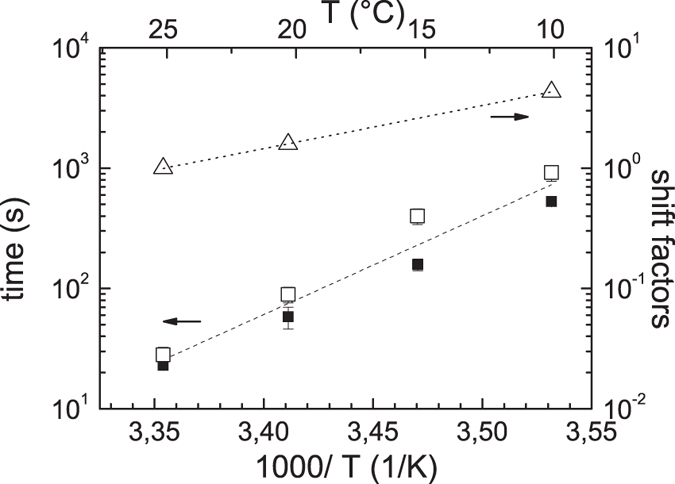
Comparison of the different timescales. Self-healing time (filled squares) and terminal relaxation time (open squares) versus inverse temperature. A common Arrhenius temperature dependence (fit) is shown as a dashed line. Also included are the shift factors for homopolymer PIB for comparison (open triangles). The corresponding Arrhenius dependence is shown as a dotted line.

A nonlinear shear deformation driven by high shear rates was chosen to prepare a well-defined damaged, out-of-equilibrium state of the network. The subsequent recovery of the network allows characterization of the dynamics of self-healing. Fig. 5 shows exemplary results of the first step, so-called startup shear experiments for different shear rates 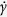 measured at 25 °C. The shear rates can be normalized with the terminal relaxation time *τ* obtained from linear rheology experiments giving the Weissenberg number 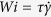. For *Wi* smaller one, the dynamic network has enough time to rearrange during deformation and the stress-strain curve shows viscoelastic behavior: an elastic linear increase at short times/small deformation and a viscous plateau at long times/large deformations. One can model this curve with a simple Maxwell model with the same parameters as for the linear dynamic measurements. For large *Wi* the stress-strain curve shows yielding, i.e. a pronounced maximum with a sharp drop at large deformations, signaling network failure. This process is related to rupture of the network within a localized fracture zone^31^. The observed dependence of the yield point on 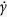 is in keeping with existing models of fracture in reversible networks, in which the lifetime of the dynamic bonds is reduced under the influence of growing stress^31^. The state after yielding was taken as a well-controlled starting point for the self-healing experiment designed as schematically depicted in the upper part of Fig. 6. The first step consists of a startup shear experiment with a high rate until time *t*_1_ beyond the yield point. At this point no further deformation is applied and a waiting period of 30 s is kept during which the stress relaxes to a large part. At the end of this period, at time *t*_2_ the deformation is manually reduced by a small amount to reach a state of approximately zero stress. Now a dynamic measurement with a small amplitude and fixed frequency is started to follow the recovery of the elastic modulus as a function of time^31^,^32^. The real part of the dynamic modulus is used to monitor the reestablishment of the disrupted network after yielding. The result of a series of experiments performed at different temperatures is shown in the lower part of Fig. 6. It shows the elastic modulus at 10 rad/s, normalized to the final value reached within the experiment as a function of time (final value within 87–93% of the equilibrium value, non-normalized data shown as Supplementary Fig. S1). A steep initial rise in the beginning is followed by a much slower perfectioning process responsible for about the last 5% of the recovery. Measurements at even shorter times than shown were not feasible, because the still ongoing stress relaxation from the former part of the experiment disturbs the small oscillatory torque signal of the recovery experiment. The time at which the modulus reached 90% of the final value, which is still in the first fast part of the recovery process, was taken as a typical time of self-healing. This value is estimated for different measurement temperatures from 25 °C down to 5 °C. The result is shown in Fig. 7 which in addition for comparison also shows the terminal relaxation time and the shift factors of PIB homopolymers as a function of inverse temperature. Clearly, self-healing time and terminal relaxation time are very similar in value and even more importantly have the same temperature dependence, i.e. they are related to the same molecular process. The chain dynamics on the other hand shows a different temperature dependence. The lines in Fig. 7 correspond to an Arrhenius law with activation energies of 157 kJ/mol for the self-healing time/terminal relaxation time and 65 kJ/mol for the chain dynamics. The latter was estimated from the temperature dependence of the shift factors for the master curve construction for the homopolymer.

## Discussion

Our results give a clear picture of the relaxation process underlying self-healing in the investigated supramolecular polymer network and furthermore allow to formulate some general characteristics and design rules for these type of materials. First of all, the formation of the network relies on a combination of hydrogen-bonding and microphase separation. Hereby the barrier for the exchange of chain ends between different micelles determines the time scale of the terminal relaxation time which sets also the time scale for self-healing. As a requirement, chain dynamics itself has to be much faster to supply the necessary mobility. Accordingly, there is a certain temperature dependent frequency window between the terminal relaxation and the tail of the Rouse dynamics in which the material behaves like a rubber with a plateau in the real part of the dynamic modulus. The value of the modulus in such systems depends on the network density which for the material investigated here is given by the number density of aggregates, i.e. basically by the molecular weight. In consequence, for supramolecular polymer networks the self-healing kinetics and the modulus of the resulting network can be tuned independently by the selection of the functional groups and by the molecular weight of the backbone chain. Details of this relation will be the topic of further experimental studies. So, although there is some similarity in the general shape of supramolecular networks and simple polymer melts, the two materials behave in detail quite differently. It is furthermore interesting to compare our results to similar experiments on a dilute triblock copolymer gel swollen by a selective solvent for the middle block^33^. In this system the self-healing time was about an order of magnitude larger than the relaxation time. The largely different times were explained by an entropic barrier related to re-association of dangling chains with the network junctions. Obviously the high concentration of the network junctions reduces this barrier in the bulk system. Transport processes necessary for reassembly have also been considered in molecular models of self-healing. A model of unentangled polymer chains with stickers only at the chain end, similar to our system, was studied in ref. 8. The difference from our system is that the other chain end is fixed to a permanent background network and that only two stickers can bind in the model. This excludes the existence of micellar aggregates present in our system. Because of the permanent network, the model will also not show terminal flow. In view of these differences an analyis of our experimental results in terms of the above mentioned theoretical model is difficult and was therefore not attempted here. In summary, we have identified the molecular process for self-healing in a supramolecular polymer network in the bulk state. Such networks consist of aggregated functional groups, bridged by polymeric backbone chains. After fracture, the dynamic exchange of functional groups between aggregates enables self-healing by reassembly of the network structure. The same process shows up in linear rheology as terminal relaxation process enabling viscous flow at long times. We anticipate, that these results on the different role of polymeric backbones and functional groups on material properties will help to design self-healing materials with specific properties. This may further broaden the scope of applications of these fascinating materials and contribute to life-cycle improvement and safety-improvement of materials.

## Methods

### Sample

Polyisobutylene (PIB) was synthesized and afterwards functionalized at both chain ends with barbituric-acid groups (see Fig. 1)^20^. The molecular weight was in the range of the critical entanglement molecular weight for PIB (*M*_*c*_ = 13.1 kg/mol)^34^. A PIB homopolymer was used to determine the shift factors as measured on a rheological experiment. Table 1 gives information about the molecular weight and defines the abbreviations for the sample names. More detailed information about the synthesis and characterization of the sample including the demonstration of self-healing ability in a conventional ‘tear-and-glue’ experiment can be found in ref. 20.

### Rheology

All rheological measurements were performed using an Anton Paar MCR 501 rheometer. A parallel plate geometry with a diameter of 8 mm was used for the shear measurements. The sample thickness was typically chosen between 0.3 mm and 0.5 mm. Temperature was controlled by the lower Peltier plate and/or streaming nitrogen gas inside the sample chamber. The parallel plate geometry was also used for the nonlinear rheological measurements, although this causes a nonuniform deformation amplitude within the sample and is only a compromise compared to a cone and plate geometry. But this approach allowed us to adapt the gap to the very limited amount of sample available.

At first, strain sweep experiments at a fixed angular frequency were performed to find the limit for linear response behavior. Only afterwards regular linear rheological measurements covering a temperature range from 120 °C to around −20 °C and a wide range of angular frequencies were performed. After each change in temperature an equilibration time of at least 10 min was introduced. Repeated measurements in cooling or heating confirmed the reproducibility of the results.

Nonlinear rheological measurements were performed as startup shear experiments with different shear rates. After each experiment, the sample was heated up to 120 °C to erase the history and reshape the disturbed sample geometry. Before starting the next measurement, an equilibration time at the new temperature of at least 15 min was kept.

### SAXS

Small angle X-ray scattering (SAXS) experiments were performed in house with a setup consisting of a Rigaku rotating anode, a focusing X-ray optics from Osmic and a Bruker 2D-detector. Cu K_*α*_ radiation with a wavelength of *λ* = 1.54 Å was used. The sample holder is an aluminium disc with a hole in the middle of 1 mm diameter. The sample was placed into the hole, and the aluminum disc attached onto a Linkam hot stage TMS 94 by thermal conducting paste. Scattering vector values *q* in the range from 0.04 Å^−1^ to 0.4 Å^−1^ were measured. The experiments were done at a series of temperatures from −40 °C to 120 °C in heating and cooling scans.

## Additional Information

**How to cite this article**: Yan, T. *et al*. Unveiling the molecular mechanism of self-healing in a telechelic, supramolecular polymer network. *Sci. Rep.* **6**, 32356; doi: 10.1038/srep32356 (2016).

## Supplementary Material

Supplementary Information

## Figures and Tables

**Table 1:** Sample characteristics.

Samples		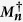 [kg mol^−1^]	*PDI*^‡^
Telechelic polymer	PIB14K-BA2	14/13.8	1.2
homopolymer	PIB30K	30	

^†^Molecular weights obtained by NMR/GPC.

^‡^Polydispersity index *PDI* = *M*_*w*_ /*M*_*n*_ as obtained by GPC.
