# Supplementary material for: Unveiling the molecular mechanism of self-healing in a telechelic, supramolecular polymer network
Source: Sci Rep. 2016 Sep 1;6:32356. doi: 10.1038/srep32356 (PMC5007665; doi:10.1038/srep32356)
Supplement: Supplementary Information [file srep32356-s1.pdf]

Supporting Information: Unveiling the molecular mechanism of self-healing in a telechelic, supramolecular polymer network

Tingzi Yan,[1] Klaus Schröter,[1] Florian Herbst,[2]
Wolfgang H. Binder,[2] and Thomas Thurn-Albrecht[1,*]

[1] *Experimental Polymer Physics, Institute of Physics,
Martin Luther University Halle-Wittenberg, Halle 06120, Germany*

[2] *Chair of Macromolecular Chemistry, Institute of Chemistry,
Martin Luther University Halle-Wittenberg, Halle 06120, Germany*

(Dated: July 15, 2016)

* thomas.thurn-albrecht@physik.uni-halle.de

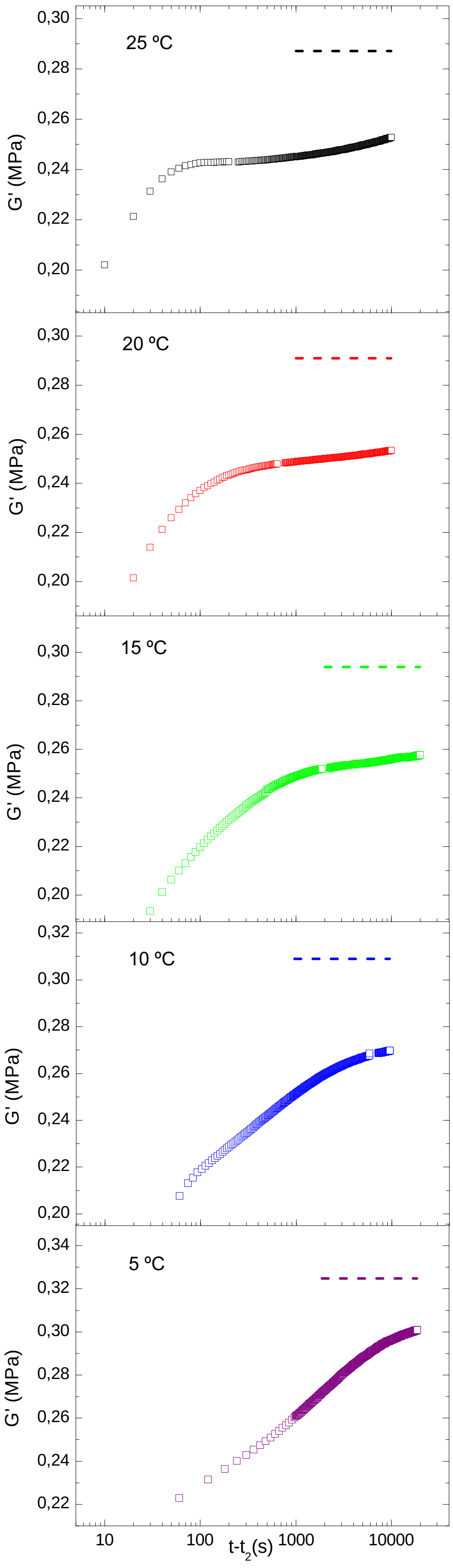

Figure S1. (Caption next page.)

Figure S1. (Previous page.) Storage modulus during recovery for sample PIB14K-BA2 at different temperatures as indicated. The data are identical to the data shown in Fig. 6 of the manuscript, but not normalized. The dashed lines denote the modulus measured prior to the nonlinear startup shear. All measuremens were performed with 10 rad/s. For each data set the ratio between the lower-end value and the upper-end value of the vertical axis is identical (=0.6) to enable a comparison with the normalized data shown in Fig. 6.
